# Anionic Anchoring Enhanced Quasi Solid Composite Polymer Electrolytes for High Performance Lithium Metal Battery

**DOI:** 10.3390/polym15244716

**Published:** 2023-12-15

**Authors:** Ruliang Liu, Xinyi Lai, Jiaqi Xue, Haiping Chen, Lijun Xie, Yanxuan Qiu, Wei Yin

**Affiliations:** School of Chemistry and Materials Science, Guangdong University of Education, Guangzhou 510303, China; 13798943464@163.com (X.L.); 17652806905@163.com (J.X.); 18319168585@163.com (H.C.); qiuyanxuan@gdei.edu.cn (Y.Q.); yinwei@gdei.edu.cn (W.Y.)

**Keywords:** lithium metal batteries, poly (vinylidene fluoride-co-hexafluoropropylene) (PVDF-HFP), zeolitic imidazole framework-8 (ZIF-8), composite polymer electrolytes

## Abstract

Herein, ZIF-8 inorganic particles with different sized reinforced poly (vinylidene fluoride-co-hexafluoropropylene) (PVDF-HFP) solid composite polymer electrolytes (PVDF-HFP/10%ZIF-8) were prepared via a facile blade-coating approach, and free-standing quasi solid-state composite electrolytes (PVDF-HFP/10%ZIF-8(0.6)/Plasticizer, abbreviated as PH/10%ZIF-8(0.6)/P), were further obtained through the introduction of plasticizer. Optimized PH/10%ZIF-8(0.6)/P exhibited a high ionic conductivity of 2.8 × 10^−4^ S cm^−1^ at 30 °C, and superior Li^+^ transfer number of 0.89 with an ultrathin thickness (26 µm). Therefore, PH/10%ZIF-8(0.6)/P could effectively inhibit the growth of lithium dendrites, and the assembled Li/LiFePO_4_ cell delivered good cycling stability with a capacity retention rate of 89.1% after 100 cycles at 0.5 C.

## 1. Introduction

At present, regarding the continuous growth of energy demand, storage devices with higher energy density have become one of the pursuits of modern society. At present, the anode materials of lithium-ion batteries, such as graphite, have an energy density of only 372 mAh g^−1^, which limits the further development of high energy density energy storage batteries [1]. Lithium metal is considered to be one of the ideal anode materials for next-generation high-energy-density batteries because of its low density (0.534 g cm^−3^), ultrahigh theoretical specific capacity (3860 mA h g^−1^), and ultralow standard reduction potential (−3.04 V vs. SHE) [2]. However, lithium metal batteries (LBMs) still face some challenges, such as uncontrolled growth of lithium dendrites and flammable liquid electrolytes. In order to improve the security and cycling stability of LMBs, developing solid electrolytes (SEs) to substitute liquid electrolytes is considered to be a highly effective strategy to address the aforementioned bottleneck issues [3]. According to the chemical components of SEs, SEs are usually classified as inorganic solid electrolytes, solid polymer electrolytes, and solid composite electrolytes [4,5,6].

Inorganic solid electrolytes (ISEs) have high ionic conductivity, wide electrochemical stability window, excellent thermal stability, and mechanical properties. However, rigid ceramic electrolytes have poor contact with electrodes, making it difficult to buffer the volume changes of positive and negative electrode materials during cycling. When assembling cells with high-voltage positive electrodes, side reactions can easily occur at the interface, seriously affecting the dynamic reaction of the battery [7]. Flexible solid polymer electrolytes (SPEs), composed of a uniform mixture of polymer substrates and lithium salts, can provide mechanical support for batteries, and process low cost, high processability, and good interface compatibility. So far, several polymers have been developed, such as polyethylene oxide (PEO), polytrimethylene carbonate (PTMC), polypropylene carbonate (PPC), and poly (vinylidene fluoride-co-hexafluoropropylene) (PVDF-HFP), and are the hosts of solid polymer electrolytes [8]. The conductivity of solid polymer electrolytes is much lower than that of commercial liquid electrolytes (e.g., 1 M LiPF_6_ in ethylene carbonate and dimethyl carbonate (1:1 by volume)), which greatly limits the widespread application of solid polymer electrolytes [9,10]. In addition, due to the low elastic modulus of the polymer matrix, the thermal and mechanical properties of the solid polymer electrolytes are poor, which cannot completely prevent the penetration of lithium dendrites and effectively ensure the safety of the battery.

In recent years, the probability of composite polymer electrolytes (CPEs) has been proposed to address the issues. A composite solid electrolyte is usually composed of inorganic materials (nanoparticles, nanofibers, nanosheets, aerogels, or vertically arranged porous structures) dispersed in a solid polymer electrolyte [11,12,13]. Further modification of inorganic ceramic fillers can effectively solve interface problems and promote uniform dispersion of inorganic fillers in polymer matrices. CPEs have the advantages of both pure inorganic ceramics, as well as pure organic polymer electrolytes, exhibiting excellent flexibility, high ionic conductivity, and good contact with electrodes [14,15,16,17,18]. Moreover, as the composite solid electrolyte inherits the flexibility and processability of the polymer matrix, it can adapt to the large-scale manufacturing process of traditional lithium-ion batteries [19]. Many efforts have been performed to resolve these issues. For example, Zhang et al. reported that functionalization modification was carried out on the surface of sulfide ceramic fillers, and the prepared composite solid electrolyte could self-repair fractures caused by lithium plating during cycling, preventing interface growth and lithium wire penetration [20]. Chen et al. constructed a high-performance poly (vinylidene fluoride)/Li_6.4_Ga_0.2_La_3_Zr_2_O_12_/succinonitrile (PVDF/LLZO/SN) composite solid electrolyte. Profiting from good interfacial stability and strong coordination effect, PVDF/LLZO/SN enabled the battery to operate at rates up to 6 C [21]. Wen et al. designed a type of ceramic particle molecular brush (MB-LLZTO) through a surface grafting strategy. The combination of the molecular brush and PEO changed the Li^+^ diffusion pathway, and the fast ion conduction ionic conductivity on the LLZTO surface increased by an order of magnitude [22]. 

Despite the recent progress on reducing the interfacial resistance between fillers and polymers in CPEs, there are still some challenges. On the one hand, due to the limited intermolecular free volume used for Li^+^ storage and transportation in linear polymers and inorganic fillers, there is poor ion transport efficiency. On the other hand, there is inevitably a “point to point” interface contact between CPEs and lithium negative electrodes, which cannot adapt to the volume changes of lithium negative electrodes during the cycling process, resulting in poor interface contact and uneven lithium metal deposition [23,24]. Therefore, constructing high-performance, solid-state lithium metal batteries with excellently long cycle life remains a challenge. In comparison to traditional ceramics with non-pore and high-mass density, metal organic frameworks (MOFs) have rich channels, plenty of open metal sites, and a high specific surface area, which can provide favorable pathways for rapid ion conduction, as well as improve ion transport between solid phase interfaces. Therefore, MOFs have begun to be applied in CPEs and have received widespread attention [25,26,27,28].

**Figure 1:**
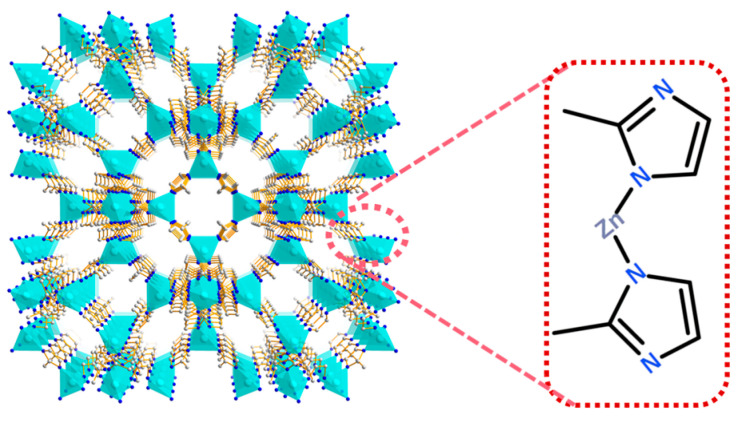
Schematic illustration for the architecture of ZIF-8.

**Scheme 1:**
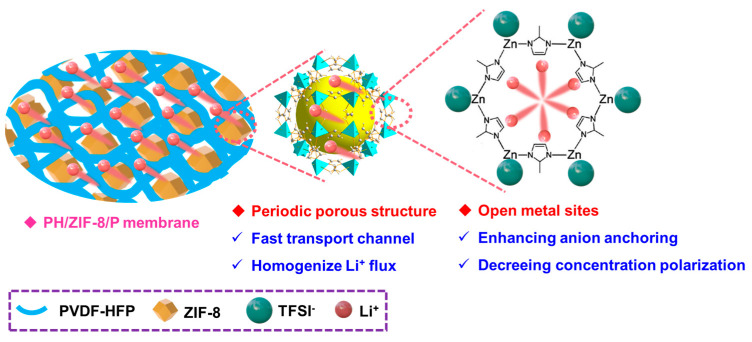
Schematic illustration for the architecture of the self-standing quasi solid composite polymer electrolytes (e.g., PH/10%ZIF-8(0.6)/P) membrane with a magnification showing nano-channels of ZIF-8. The migrating Li^+^ are highlighted by the stretched red spheres.

Among these MOFs, the zeolitic imidazole framework-8 (ZIF-8) (Figure 1) is considered a promising functional filler for solid electrolytes due to not only inheriting the advantages of MOFs, but also having excellent chemical and thermal stability. Herein, we designed a novel quasi solid CPE, which is composed of ZIF-8, PVDF-HFP, lithium salt (lithium bis(trifluoromethylsulphonyl) imide, LiTFSI), and plasticizer (fluorinated ethylene carbonate, FEC). Such quasi solid CPEs showed high lithium-ionic conductivity, and a high lithium-ion transference number that enabled LMBs with excellent rate performance and long cycling stability. ZIF-8 crystals of different sizes were first synthesized under different reaction conditions. Subsequently, they were uniformly mixed with lithium salts and polymer matrices (PVDF-HFP, abbreviated as PH), and through a simple and efficient scraping process, an ultra-thin and flexible CPE was obtained (PH/ZIF-8). Finally, a small amount of FEC as plasticizer (21 wt%) was introduced to prepare a novel quasi solid CPE (PH/10% ZIF-8/Plasticizer, abbreviated as PH/10%ZIF-8(0.6)/P). Compared with traditional quasi solid CPEs, the resulted PH/0% ZIF-8/P has the following characteristics (Scheme 1). (i) ZIF-8 has a unique porous structure and a large number of open metal sites, which are conducive to fixing lithium salt anions and promoting rapid transport of lithium ions, thereby increasing the transference number of lithium ions. (ii) PVDF-HFP with a high dielectric constant is conducive to the rapid dissociation of lithium salts and improves ionic conductivity. (iii) The introduction of plasticizers can enhance interface compatibility while assisting in the construction of stable and sturdy solid electrolyte interface (SEI) layers, effectively inhibiting the growth of lithium dendrites, and achieving uniform deposition of lithium metal.

## 2. Materials and Methods

### 2.1. Materials

Zinc acetate (Aladdin, Shanghai, China), 2-Methylimidazole (C_4_H_6_N_2_) (Aladdin, Shanghai, China), Bis (trifluoromethanesulfonyl) imide (LiTFSI) (Aladdin, Shanghai, China), Poly (vinylidene fluoride-co-hexafluoropropylene) (PVDF-HFP, Mw ≈ 400,000, Aldrich, Shanghai, China), Polyvinylidene fluoride (PVDF, Mw ≈ 534,000, Aldrich, Shanghai, China), KS Conductive Graphite (Timcal, Shanghai, China), LiFePO_4_ (LFP, Kelude, Dongguan, China), Aluminium foil (15 μm, Kelude, Dongguan, China), Lithium (0.3 mm, Kelude, Dongguan, China), N-methylpyrrolidone (NMP, Aladdin, Shanghai, China), Fluorinated ethylene carbonate (FEC, Aladdin, Shanghai, China), Methanol (Macklin, Shanghai, China), N, N-Dimethylformamide (DMF, Macklin, Shanghai, China).

### 2.2. Synthesis of ZIF-8 Nanoparticles with Different Size and PVDF-HFP/ZIF-8 Composite Polymer Electrolytes

(i) Synthesis of small-sized ZIF-8 nanoparticles (ZIF-8 (0.6) NPs)

Zinc acetate (175 mg) and 2-methylimidazole (263 mg) were mixed. Then, methanol (40 mL) was added to dissolve the mixed powder. The solution was stirred for 5 min, then the mixture was allowed to stand at room temperature for 24 h. White powder precipitate appeared, which was sufficiently washed with methanol, and dried to obtain ZIF-8 (0.6).

(ii) Synthesis of large-size ZIF-8 nanoparticles (ZIF-8 (1.3) NPs)

Zinc acetate (175 mg) was dissolved in methanol (20 mL) to form mixed solution 1. 2-Methylimidazole (263 mg) was dissolved in methanol (20 mL) to form another mixed solution 2. The two solutions were then mixed and stirred for 5 min. The mixture was allowed to stand at room temperature for 24 h. A white powdery precipitate was obtained, which was washed thoroughly with methanol, and dried to obtain ZIF-8 (1.3).

(iii) Preparation of PVDF-HFP/ZIF-8 composite polymer electrolytes

150 mg of PVDF-HFP, 75 mg of LiTFSI, and 1.2 mL of DMF were added to the bottle, and then ZIF-8 (0.6), which accounted for 10%, 20%, 30%, and 40% of the mass of the polymer PVDF-HFP, was added to the bottle sequentially, and the homogeneous slurry was obtained by stirring overnight. The slurry was casted onto a glass plate with a doctor blade, and then dried in a vacuum oven at 70 °C for 12 h to obtain PVDF-HFP/x%ZIF-8(0.6) composite polymer electrolytes (PH/x%ZIF-8(0.6), x = 10, 20, 30 or 40). PVDF-HFP/x%ZIF-8(1.3) composite polymer electrolytes (PH/x%ZIF-8(1.3), x = 10, 20, 30 or 40) were prepared via the same process except for using ZIF-8(1.3) instead of ZIF-8(0.6).

### 2.3. Preparation of Cathode and Battery Assembly

LFP, PVDF, and conductive graphite were mixed in an agate mortar according to the mass ratio of 8:1:1. NMP was added dropwise to obtain homogenous slurry by grinding for 1 h. Finally, the carbon-coated aluminum foil was coated with a silica gel brush, then scraped on a coating machine, and then dried in a vacuum at 60 °C for 12 h. The slurry was applied to a carbon-coated aluminum foil. After drying, it was tailored into round disks with a 12 mm diameter. Li/Li symmetric cells and Li/LFP cells were assembled in the argon-filled glove box. For the PH/x%ZIF-8 composite polymer electrolyte series samples, 5 µL plasticizer (FEC) was added dropwise to assemble quasi solid-state cells.

### 2.4. Characterizations

Morphologies were investigated by field-emission scanning electron microscopy (SEM, TESCAN MIRA LMU, Brno, Czech Republic). X-ray diffraction (XRD, Bruker, Billerica, MA, USA) patterns were achieved with a D-MAX 2200 VPC X-ray diffractometer with Ni-filtered Cu/K-α radiation at a scan rate of 10° min^−1^ from 10° to 80°. The thermogravimetric analysis (TGA) of membranes was carried out using the TGA 4000 instrument (Thermo Fisher Scientific, Waltham, MA, USA) at a heating rate of 10 °C min^−1^, and in a nitrogen atmosphere. Cyclic voltammetry (CV) and electrochemical impedance spectroscopy (EIS) were tested using an electrochemical workstation (Coster CS350, Wuhan, China). Linear sweep voltammetry (LSV) was scanned at a speed of 1 mV s^−1^ (voltage range, 0–6 V), and EIS was scanned at an AC voltage amplitude of 5 mV between 100 kHz and 0.1 Hz at various temperatures from 30 to 90 °C. The galvanostatic charge-discharge test was recorded using the NEWARE Battery Test System (Shenzhen Neware Electronics Co., Ltd., Shenzhen, China) at a voltage range of 2.5–4 V (vs. Li/Li^+)^ with the calculated C-rate based on the theoretical capacity value of LFP (170 mA g^−1^)). Cycling and rate performances were tested at 25 °C.

The ionic conductivity was calculated according to the following Equation (1):

σ = *L*/*SR* (1)

where *L* is membrane thickness, *S* is electrode area, and *R* is bulk resistance obtained from the EIS data.

Li^+^ transference number ($t_{{Li}^{+}}$) was calculated based on the following Equation (2):

(2) $$t_{{Li}^{+}}=\frac{I_{S}(∆V-I_{0}R_{0})}{I_{0}(∆V-I_{S}R_{S})}$$

where ∆*V* is polarization voltage, *I*_0_ and *I_s_* are the initial and steady-state currents, respectively, *R*_0_ and *R_s_* are the initial and steady-state resistances obtained from the EIS data, respectively. The testing temperature is 30 °C, and the polarization voltage is 10 mV.

## 3. Results and Discussion

**Figure 2:**
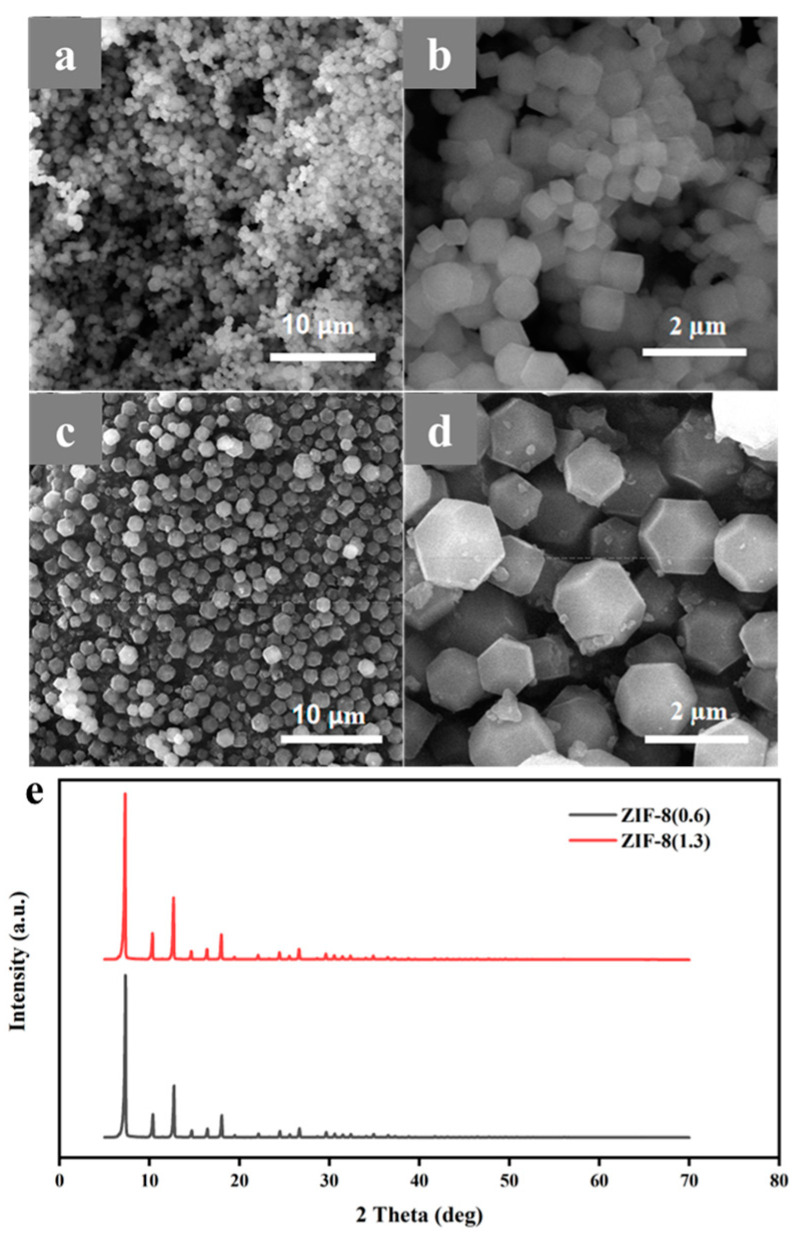
SEM images of (**a**,**b**) ZIF-8 (0.6) and (**c**,**d**) ZIF-8 (1.3), (**e**) XRD curves of ZIF-8 (0.6) and ZIF-8 (1.3) powder.

The microstructure of as synthesized ZIF-8 was firstly observed through the scanning electron microscope (SEM). As shown in Figure 2a–d, ZIF-8 nanoparticles show different sizes when using different Zinc salt. It can be observed that under the same magnification, the average sizes of two ZIF-8 are approximately 0.6 and 1.3 µm, respectively, named ZIF-8 (0.6) and ZIF-8 (1.3). Meanwhile, the enlarged image of ZIF-8 shows that the microstructures of ZIF-8 (0.6) (Figure 2b) and ZIF-8 (1.3) (Figure 2d) are both regular dodecahedrons, which is consistent with the reported literatures [29,30]. X-ray diffraction (XRD) patterns of ZIF-8 (0.6) and ZIF-8 (1.3) are shown in Figure 2e. The characteristic peaks at 7.3°, 10.3°, 12.7°, 14.7°, 16.4°, and 18.0°, are attributed to the (011), (002), (112), (022), (013), and (222) crystal planes of ZIF-8, respectively, indicating ZIF-8 was successful synthesized [31]. To investigate the pore structure of ZIF-8, the adsorption desorption isotherm of N_2_ was carried out. The Brunauer-Emmett-Teller (BET) surface area and average pore diameter were calibrated to be 1556 m^2^ g^−1^ and 1.55 nm, respectively (Figure S1). Abundant microporous structures can provide more storage space for lithium ions, which is conducive to rapid transport of lithium ions. 

**Figure 3:**
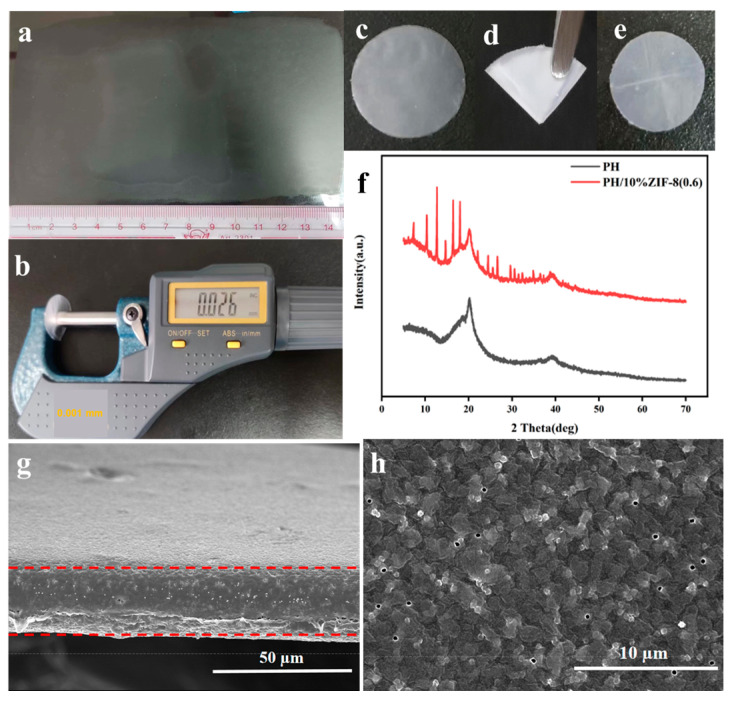
(**a**) Digital photos of macro scale PH/10%ZIF-8(0.6) membrane and (**b**) thickness measurement of PH/10%ZIF-8(0.6) membrane, (**c**) Digital photos of free-standing PH/10%ZIF-8(0.6) membrane and (**d**,**e**) bending PH/10%ZIF-8(0.6) membrane, (**f**) XRD curves of PH and PH/10%ZIF-8(0.6) membrane. (**g**) Cross-section and (**h**) surface SEM images of PH/10%ZIF-8(0.6) membrane (The red dashed line represents the cross-sectional boundary of the membrane).

Macro scale PVDF-HFP/ZIF-8 (0.6) (PH/ZIF-8 (0.6)) CPE membrane can be obtained via a simple and efficient scraping process (Figure 3a). The average thickness of the prepared PH/ZIF-8 (0.6) CPE is as low as 26 µm (Figure 3b). As shown in Figure 3c–e, the PH/ZIF-8 (0.6) CPE membrane can still maintain a complete macroscopic morphology after repeating bending and folding 200 times, indicating that the PH/ZIF-8(0.6) CPE has good flexibility. XRD of the PH/ZIF-8(0.6) and PH membrane are shown in Figure 3f. After introducing ZIF-8, the characteristic diffraction peak intensity of PH showed a certain degree of weakening, indicating that ZIF-8 fillers can reduce the crystallization of polymers, which is favorable for lithium-ion transport. SEM characterization of PH/ZIF-8 (0.6) CPEs was also conducted. The PH/ZIF-8 (0.6) CPE has a uniform thickness (26 µm) according to the cross-sectional SEM image (Figure 3g), which is consistent with the film thickness measured by calipers. As shown in Figure 3h, ZIF-8 nanoparticles are more evenly dispersed on the surface of PH/ZIF-8 (0.6) CPE membrane. Meanwhile, a number of irregular micron scale pores can be observed, which is conducive to promoting the efficiency of lithium-ion transport. 

**Figure 4:**
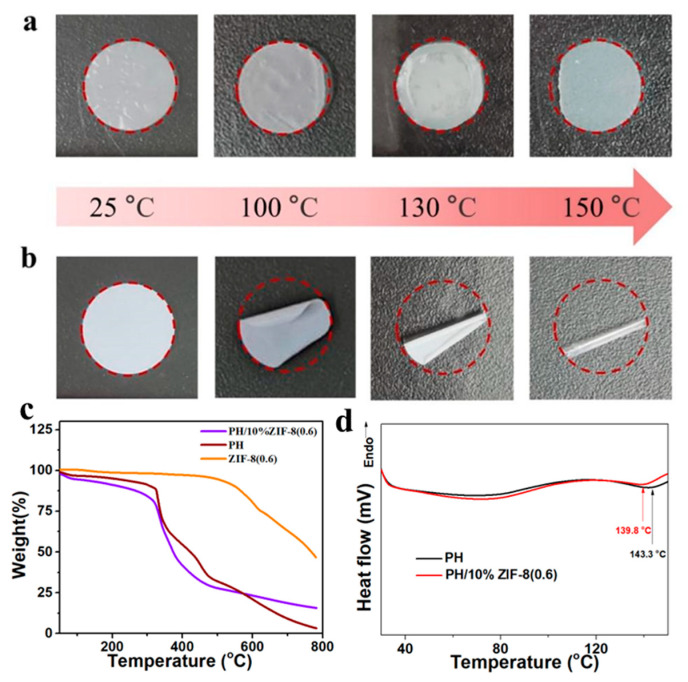
Thermal shrinkage of (**a**) PH/10%ZIF-8(0.6) membrane and (**b**) PP membrane after heating at different temperatures. (**c**) TGA curves of PH/10%ZIF-8(0.6) membrane, PH membrane and ZIF-8(0.6). (**d**) DSC curves of PH/10%ZIF-8(0.6) and PH membrane.

Thermal stability of solid-state electrolyte membranes is seriously affecting the safety performance of LMBs. The PH/10% ZIF-8 (0.6) membrane and commercial polypropylene (PP) membrane were placed in an oven and maintained at different temperatures for 0.5 h, respectively. As shown in Figure 4a, during the entire heating process (25–150 °C), the PH/10% ZIF-8 (0.6) membrane kept the integrity of the macro structure, with only slight shrinkage appearing. In sharp contrast, the PP membrane had already started to curl at 100 °C. When the temperature continued to rise to 150 °C, the PP membrane became completely curled, and the color changed from milky white to transparent (Figure 4b). Moreover, the thermal shrinkage rate of the PH/10%ZIF-8(0.6) membrane at 200 °C was as low as 3.8%, much lower than that of the PP separator (88.6%). The Thermal resistance results indicate that our PH/10% ZIF-8 (0.6) membrane has excellent thermal stability. In addition, TGA tests were conducted to investigate the thermal behavior of membranes. As depicted in Figure 4c, the decomposition temperature of PH and ZIF-8 (0.6) is 300 and 550 °C, respectively. The PH/10% ZIF-8 (0.6) membrane inherited this thermal stability well, and its decomposition temperature could still be maintained at around 300 °C. DSC curves are displayed in Figure 4d, the melting temperature of the PH/10% ZIF-8 (0.6) membrane is 139.8 °C, which is lower than that of the PH membrane (143.3 °C) (Figure 4d), confirming that ZIF-8 fillers can reduce the crystallization of polymers [32,33].

**Figure 5:**
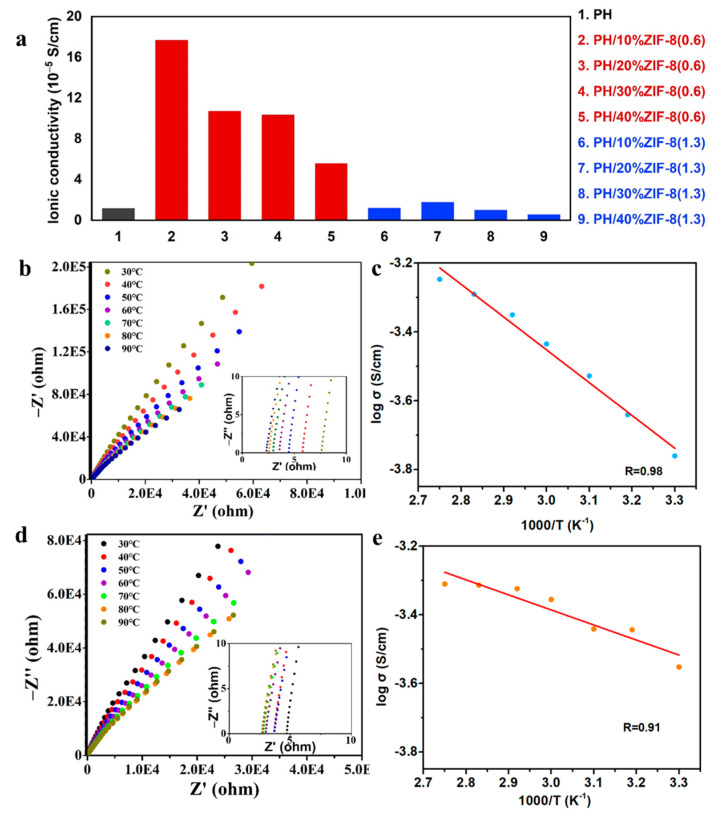
(**a**) The ionic conductivity of PH membrane, PH/x%ZIF-8(1.3) membrane and PH/x%ZIF-8(0.6) membrane at 30 °C. Impedance spectra of (**b**) PH/P membrane and (**d**) PH/10%ZIF-8(0.6)/P membrane at temperature of range 30–90 °C (inset: enlarged impedance spectra). The ionic conductivities of (**c**) PH/P membrane and (**e**) PH/10%ZIF-8(0.6)/P membrane as a function of temperature. The plots represent the experimental data and the solid lines represent fitting results.

The ionic conductivity of CPEs have a critical role in improving the performances of LMBs. Stainless steel/stainless steel (SS/SS) symmetrical cell with different CPEs were assembled to test their electrochemical impedance spectra (EIS) at 30 °C. As shown in Figure 5a, as well as in the EIS spectra of Figure S2, the ionic conductivity of the PH polymer membrane is relatively low, only 1.17 × 10^−5^ S cm^−1^ at 30 °C. When 20%ZIF-8(1.3) was added, the prepared PH/20%ZIF-8(1.3) CPEs had the best ionic conductivity (1.75 × 10^−5^ S cm^−1^) at 30 °C. And as the content of ZIF-8(1.3) increased, the impedance of the PH/x%ZIF-8(1.3) CPEs gradually increased, leading to a decrease in ionic conductivity. On the other hand, when 10 wt% ZIF-8(0.6) was added, the corresponding PH/10%ZIF-8(0.6) CPEs exhibited the highest ionic conductivity (1.76 × 10^−4^ S cm^−1^) at 30 °C. As the additional amount of ZIF-8(0.6) increased, the ionic conductivity of PH/x%ZIF-8(0.6) gradually decreased. To improve the ionic conductivity of PH/10%ZIF-8(0.6) CPEs, the corresponding quasi-solid CPEs (PH/10%ZIF-8(0.6)/P) were prepared by adding plasticizer. For PH/10%ZIF-8(0.6) and PH/10% ZIF-8(0.6)/P, the dependence of the logarithm of conductivity on the inverse temperature has been explored. As shown in Figure 5b,d, their impedance continuously decreased with the increasing temperature, and the ionic conductivity of PH/10%ZIF-8(0.6)/P was up to 2.8 × 10^−4^ S cm^−1^ at 30 °C after introducing 21 wt% plasticizer into the composite membrane. Meanwhile, the relationship between the ionic conductivity and temperature of PH/10%ZIF-8(0.6) (Figure 5c) and PH/10%ZIF-8(0.6)/P (Figure 5e) followed the typical Arrhenius linear equation. According to the fitting results, the activation energy of PH/10%ZIF-8(0.6) and PH/10%ZIF-8(0.6)/P was 7.92 and 3.65 KJ mol^−1^, respectively, indicating that the introduction of plasticizers helped to reduce the activation energy and improve the ion transport efficiency.

**Figure 6:**
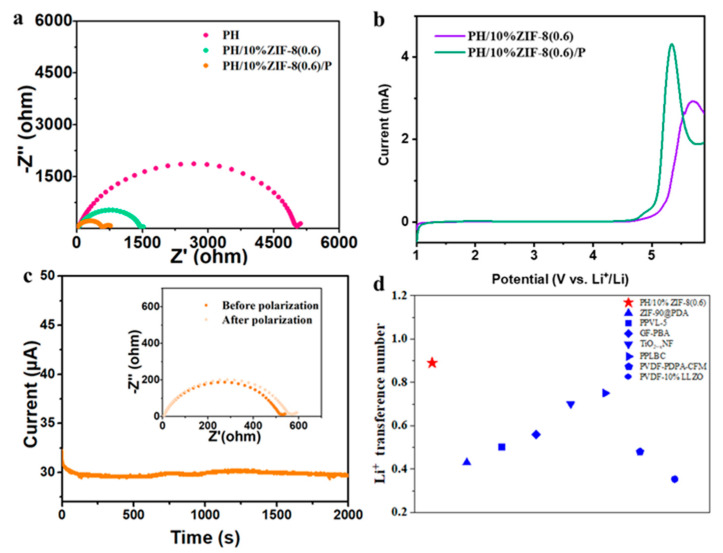
(**a**) Impedance spectra of symmetric cells with PH, PH/10%ZIF-8(0.6) membrane, and PH/10%ZIF-8(0.6)/P membrane. (**b**) LSV curves for PH/10%ZIF-8(0.6) membrane, and PH/10%ZIF-8(0.6)/P membrane at a scan rate of 1 mV s^−1^. (**c**) Chronoamperometry profiles of Li/Li symmetric cell with PH/10%ZIF-8(0.6)/P membrane at 10 mV of polarization (inset: impedance spectra before and after polarization). (**d**) Comparison of PH/10%ZIF-8(0.6)/P with the reported SEs in terms of Li^+^ transference number.

Li/Li symmetric cells based on different solid-state electrolytes were assembled to investigate the electrolyte/electrode interface reaction impedance (Rct). As shown in Figure 6a, compared to PH, the Rct of PH/10%ZIF-8(0.6) was 1460 Ω, which was much lower than that of PH (5000 Ω). After introducing a small amount of plasticizer, *Rct* of PH/10%ZIF-8(0.6) was as low as 560 Ω, demonstrating the interfacial compatibility of PH/10%ZIF-8(0.6)/P had significantly improved. The electrochemical stability window, as one of the important parameters of the electrolyte, can directly affect the matching of the positive electrode active material, thereby indirectly affecting the energy density of the battery. We assembled a series of Li/SS asymmetric batteries using FEC, PH/P, PH/10%ZIF-8(0.6), and PH/10%ZIF-8(0.6)/P as electrolytes, with Li metal and SS as counter electrodes and working electrodes, and conducted linear sweep voltammetry (LSV) testing at room temperature. The corresponding LSV curves are shown in Figure S3 and Figure 6b, the electrochemical stability window of pure FEC and PH/P was 4.6 V and 4.65 V, respectively. After introducing ZIF-8(0.6), the electrochemical stability window of the PH/10%ZIF-8(0.6) and PH/10%ZIF-8(0.6)/P was 5.0 and 4.7 V, indicating PH/10%ZIF-8(0.6)/P still had excellent electrochemical stability after the introduction of plasticizer. Our PH/10%ZIF-8(0.6)/P can match high-voltage cathodes, such as ternary NCM811 and LiCoO_2_. The high lithium-ion transference number (*t*_Li+_) can effectively alleviate the polarization effect of the electrolyte, thereby inhibiting the nucleation of lithium dendrites and slowing down the growth of lithium dendrites, thereby improving the cycle performance of LMBs. The chronoamperometry profile and the initial/steady-state EISs of Li/Li cell with solid state electrolytes are presented in Figure 6c and Figure S4, the value of *t*_Li+_ for PH/10%ZIF-8(0.6)/P was evaluated to be 0.89, which was much higher than those of PH/P (0.35) and PH (0.3). The excellent *t*_Li+_ of PH/10%ZIF-8(0.6) could be attributed to the unique 3D porous nanonetwork and rich ZnO groups in ZIF-8, which limit the movement of TFSI^−^ via chemical interaction and therefore promote mobility of Li^+^. It is worth noting that the *t*_Li+_ of our PH/10%ZIF-8(0.6)/P is also obviously higher than those of reported SEs [21,34,35,36,37,38,39] (e.g., 0.75 for PPLBC, 0.48 for PVDF-PDPA-CFM, 0.353 for PVDF-10%LLZO) (Figure 6d).

**Figure 7:**
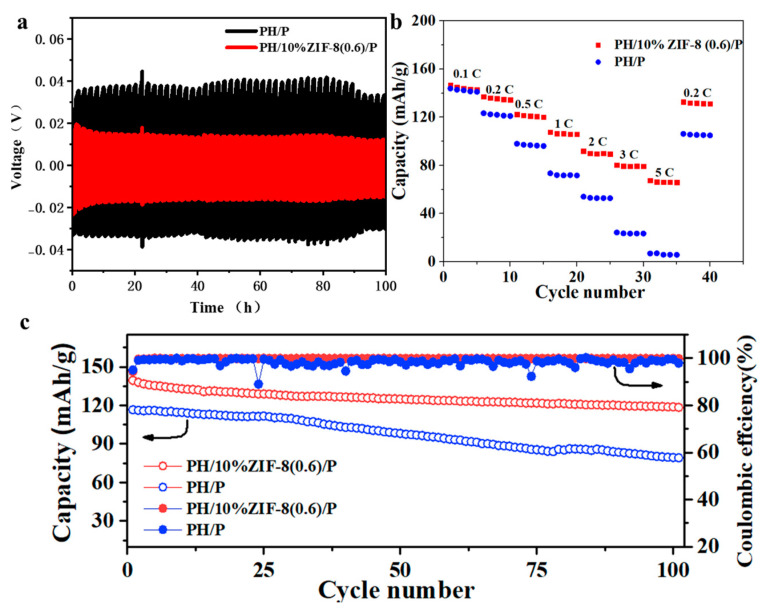
(**a**)Voltage-time profiles of Li/Li symmetric cells with PH/P and PH/10%ZIF-8(0.6)/P at 0.1 mA cm^−2^ and 0.1 mAh cm^−2^. (**b**) Rate performance of Li/LFP cell with PH/P and PH/10%ZIF-8(0.6)/P. (**c**) Cycling performance of Li/LFP cell with PH/P and PH/10%ZIF-8(0.6)/P at 0.5 C.

The polarization tests of the symmetric cells using two SEs were conducted to investigate the dynamically interfacial stability between Li metal anode and SEs. As displayed in Figure 7a, the Li|PH/10%ZIF-8(0.6)/P|Li symmetric cell exhibited a highly stable voltage hysteresis of about 18 mV during lithium plating/stripping at a capacity 0.1 mA h cm^−2^ with a current density of 0.1 mA cm^−2^, which maintained an extremely low voltage polarization without short circuiting after cycled for 100 h. In contrast, the Li|PH/P|Li symmetric cell showed larger voltage of hysteresis with random fluctuations (30–45 mV), mainly attributed to lithium dendrite growth. These results demonstrate that PH/10%ZIF-8(0.6)/P can greatly improve the cycling reversibility and stability for lithium plating/stripping in a symmetric cell. To demonstrate the potential application of PH/10%ZIF-8(0.6)/P in lithium metal full batteries, Li/LiFePO_4_ (LFP) full batteries using PH/10%ZIF-8(0.6)/P were assembled. As shown in Figure 7b, the Li|PH/10%ZIF-8(0.6)/P|LFP cell delivered specific discharge capacities of 144, 135, 121, 106, 89, and 79 mAh g^−1^ at 0.1, 0.2, 0.5, 1, 2, and 3 C, respectively. Even with increasing the current density to 5 C, the discharge capacity still maintained at 66 mAh g^−1^. When the current density was switched back to 0.2 C, a capacity of 131 mAh g^−1^ was recovered. For the Li|PH/P|LFP cell, it showed a comfortable specific capacity of 142 mA h g^−1^ and 121 mA h g^−1^ at a low current density of 0.1 C and 0.2 C, but its specific capacity decreased sharply when the current density increased, having only 6 mAh g^−1^ at a high current density of 5 C. These results shows that the Li|PH/10%ZIF-8(0.6)/P|LFP cell has a robust rate performance. In addition, Li/LFP full batteries using PH/10%ZIF-8(0.6)/P presented a high reversible capacity of 118.6 mA h g^−1^ with a capacity retention of 89.1% after 100 cycles at 0.5 C, and delivered an average coulombic efficiency 99.8%. On the contrary, the specific capacity of the Li|PH/P|LFP cell was only 78 mAh g^−1^ at the 100th cycle with a low-capacity retention of 68%. More seriously, the average coulombic efficiency of the Li|PH/P|LFP cell was only as low as 98.4%. These results reveal that our PH/10%ZIF-8(0.6)/P can greatly enhance cycling performance of LMBs.

**Figure 8:**
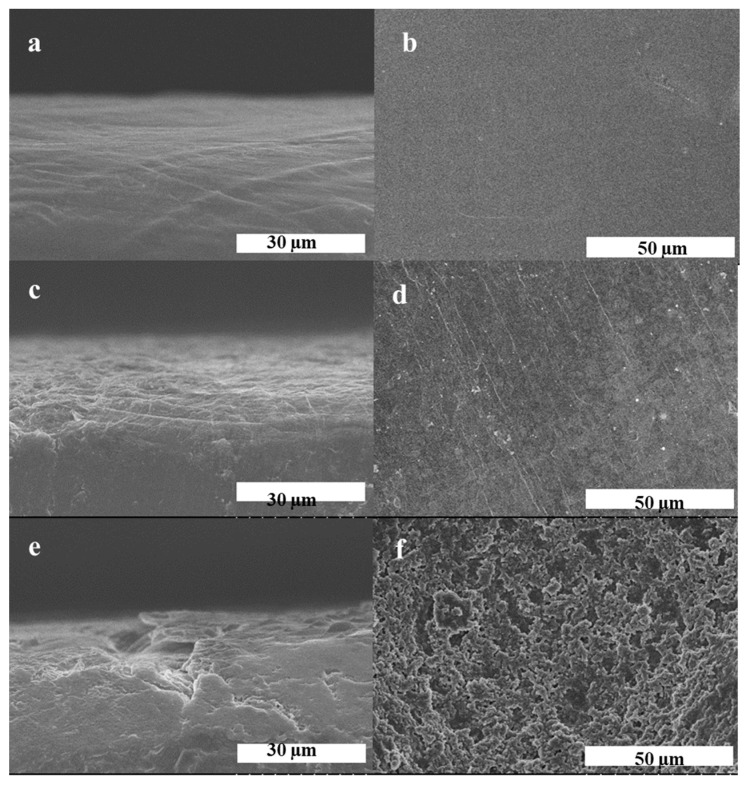
SEM images for the surface and transverse section of (**a**,**b**) the pristine Li electrode before galvanostatic cycles, and the Li electrodes obtained from the Li/Li symmetric cells with (**c**,**d**) PH/10%ZIF-8(0.6)/P and (**e**,**f**) PH/P after 50 cycles at 0.1 mA cm^−2^.

To visually investigate the suppression of PH/10%ZIF-8(0.6)/P against dendrite growth, SEM was further carried out to observe the changes of the surface and cross-section morphologies of fresh lithium metal and lithium anodes derived from cycled Li/Li symmetric cells. Fresh lithium metal showed a smooth and tight surface morphology (Figure 8a,b). After cycling, the surface of the lithium anode from the Li|PH/10%ZIF-8(0.6)/P|LFP cell remained smooth and dense without visible lithium dendrites (Figure 8c,d). In sharp contrast, the lithium anode from the cycled Li|PH/P|LFP cell showed a rough surface morphology with severe pulverization, indicating plenty of uncontrollable lithium dendrites caused due to the inevitable increase of local current density having grown on the surface of lithium anode (Figure 8e,f). These results demonstrate that PH/10%ZIF-8(0.6)/P is significantly capable of homogenizing lithium deposition and restraining lithium dendrites growth in LMBs.

## 4. Conclusions

In summary, we have developed a new quasi solid composite polymer electrolyte (PH/10%ZIF-8(0.6)/P) with improved lithium-ion transport through the introduction of ZIF-8 nanoparticles for application in LMBs. Due to the strong interaction between rich metal sites of ZIF-8 and TFSI^−^ anions, PVDF-HFP with high dielectric constant and little small molecule plasticizer, lithium-ion mobility in composite solid-state electrolytes and interface compatibility between electrodes and electrolytes are greatly improved. Furthermore, ZIF-8 nanoparticles also reinforce the thermal stability of composite solid-state electrolytes, which would be beneficial for the safety and cycle life of LMBs. The optimized PH/10%ZIF-8(0.6)/P shows enhanced ionic conductivity of 2.8 × 10^−4^ S cm^−1^ with a higher *t*_Li+_ of 0.89 at 30 °C, as well as a stable Li deposition behavior. As a result, Li/Li cells with the optimized PH/10%ZIF-8(0.6)/P CPEs display a lower polarization potential (18 mV). Li|PH/10%ZIF-8(0.6)/P|LFP cells exhibit much better cycling stability with a high-capacity retention of 89% after 100 cycles at 0.5 C, which has much lower interfacial resistance than that using PH SPEs. Therefore, this work provides a new insight to regulate the internal interactions of the compositions in CPEs through employing polymers with high dielectric constant and introducing porous framework fillers.

## Data Availability

Data are contained within the article.

## Supplementary Materials

The following supporting information can be downloaded at: https://www.mdpi.com/article/10.3390/polym15244716/s1. Figure S1. (a) N2 adsorption desorption curves of ZIF-8 (0.6) and (b) pore size distribution of ZIF-8 (0.6). Figure S2. Impedance spectra of (a) PH membrane, PH/x%ZIF-8 (1.3) membrane, and (b) PH/x%ZIF-8 (0.6) membrane at 30 °C. Figure S3. LSV curves for FEC plasticizer and PH/P at a scan rate of 1 mV s^−1^. Figure S4. Chronoamperometry profiles of Li/Li symmetric cell with (a) PH/P membrane and (b) PH membrane at 10 mV of polarization (inset: impedance spectra before and after polarization).
