# Supplementary material for: Anionic Anchoring Enhanced Quasi Solid Composite Polymer Electrolytes for High Performance Lithium Metal Battery
Source: Polymers (Basel). 2023 Dec 15;15(24):4716. doi: 10.3390/polym15244716 (PMC10748324; doi:10.3390/polym15244716)
Supplement: Supplementary file 1 [file polymers-15-04716-s001.zip › polymers-2743090-supplementary.pdf]

# Supporting information

*Article*

# Anionic Anchoring Enhanced Quasi Solid Composite Polymer Electrolytes for High Performance Lithium Metal Battery

**Ruliang Liu *, Xinyi Lai, Jiaqi Xue, Haiping Chen, Lijun Xie *, Yanxuan Qiu and Wei Yin**

School of Chemistry and Materials Science, Guangdong University of Education, Guangzhou 510303, China; 13798943464@163.com (X.L.); 17652806905@163.com (J.X.); 18319168585@163.com (H.C.); qiuyanxuan@gdei.edu.cn (Y.Q.); yinwei@gdei.edu.cn (W.Y.)
* Correspondence: liuruliang0402@163.com (R.L.); lijunxie@gdei.edu.cn (L.X.)

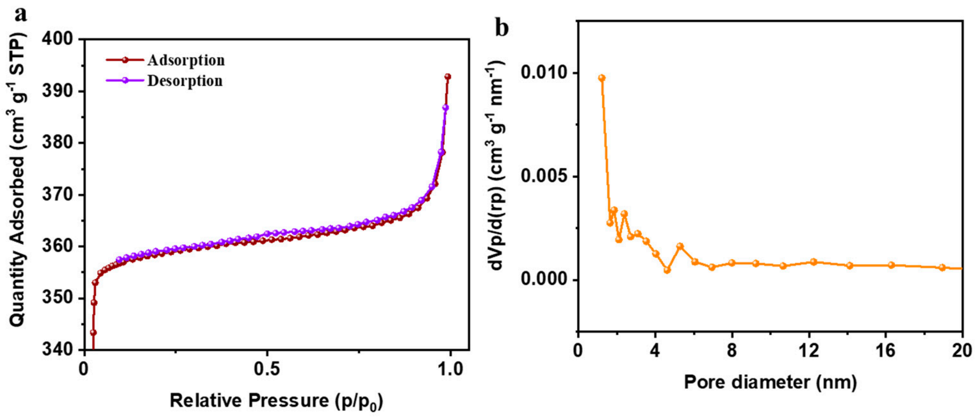


Figure S1. (a) $N_2$ adsorption desorption curves of ZIF-8 (0.6) and (b) pore size distribution of ZIF-8 (0.6).

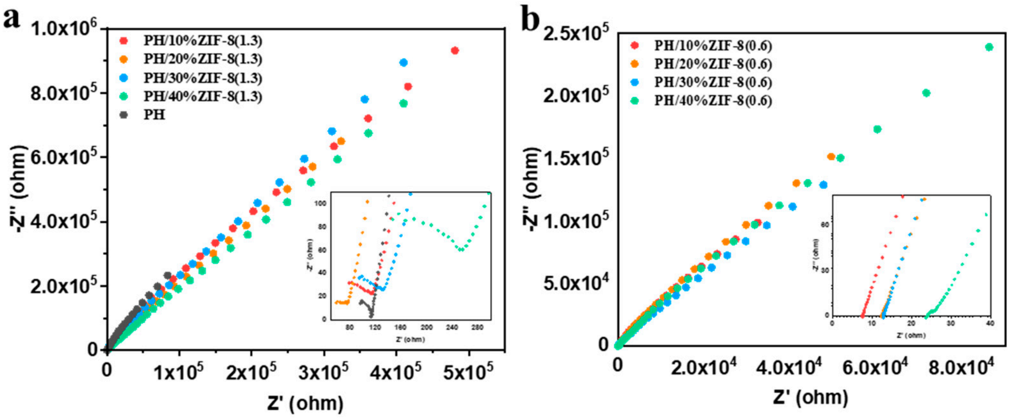


Figure S2. Impedance spectra of (a) PH membrane, PH/x%ZIF-8 (1.3) membrane, and (b) PH/x%ZIF-8 (0.6) membrane at 30 ºC.

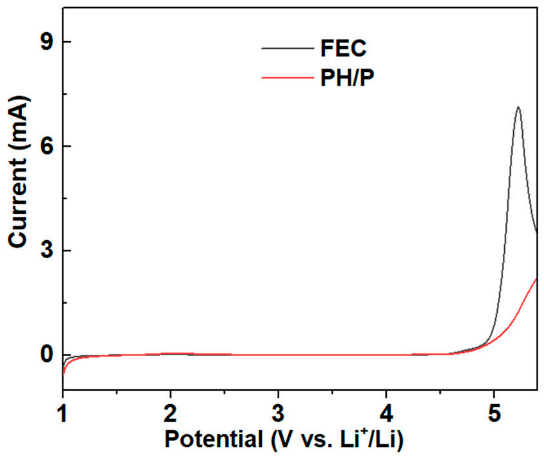


Figure S3. LSV curves for FEC plasticizer and PH/P at a scan rate of 1 mV $s^{-1}$.

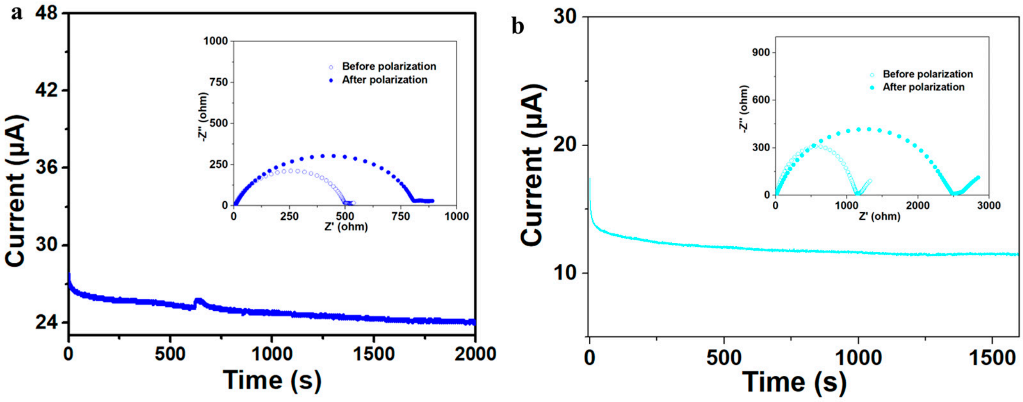


Figure S4. Chronoamperometry profiles of Li/Li symmetric cell with (a) PH/P membrane and (b) PH membrane at 10 mV of polarization (inset: impedance spectra before and after polarization).
